# Expression of Concern: *WAPO-A1* is the causal gene of the 7AL QTL for spikelet number per spike in wheat

**DOI:** 10.1371/journal.pgen.1011096

**Published:** 2023-12-20

**Authors:** The PLOS Genetics Editors

Following the publication of this article [1], concerns were raised regarding results presented in Fig 3 and the originally published S1 Data. Specifically, the corresponding author informed PLOS that the Fig 3E–3G expression data reported in this study and the corresponding Data D in S1 Data were inappropriately manipulated.

The University of California, Davis confirmed that author DPW admitted to manipulation of the data underlying the results presented in Fig 3 and S1 Data.

The authors repeated the qRT-PCR experiments presented in S1E–S1G Fig, using samples obtained from new plant materials. The results of the repeated experiments demonstrated that the transcript levels of the MADS-box genes from class-B and -C showed a highly significant downregulation in the *wapo1* mutant relative to the wildtype, and are available in the S1 File and S1 Fig files provided with this notice. These results support the conclusion reported in [1] that the downregulation of class-B and -C MADS-box genes likely contributed to the defects in anthers and pistils observed in the *wapo1* mutant.

However, in the repeated experiment data set, the *wapo1* mutant showed a marginally significant downregulation of class-E genes *SEP1-2*, *SEP1-4*, *SEP1-6* and a highly significant downregulation of *SEP3-2*, which were not reported in [1]. In light of these findings, a paragraph of the Results section and a paragraph of the Discussion section have been updated below.

In the **CRISPR *wapo1* mutant shows altered floral morphology** subsection of the Results, the second paragraph is updated to: “Since most of the floral abnormalities in *wapo1* were detected in the internal whorls (lodicules, stamens and pistils), we explored the effect of the *wapo1* mutant on the expression of class-B, -C an -E MADS-box floral genes in developing spikes at the early stamen primordia stage. Relative to the WT, the *wapo1* mutant showed a highly significant down regulation of class-B (*AP3-1* and *PI1*), class-C (*AG1* and *AG2*), and one class-E (*SEP3-2*) MADS-box genes (Fig 3E–3G). The other class-E genes, *SEP1-2*, *SEP1-4* and *SEP1-6*, showed only small and marginally significant differences (Fig 3G).”

In the **Floral and spike abnormalities in mutant and transgenic *WAPO-A1* plants** subsection of the Discussion, the first paragraph is updated to: “In addition to its role in the activation of *AP1*, LFY has been shown to bind to regulatory regions of *APETALA3* (*AP3*, a class-B floral gene) and *AGAMOUS* (*AG*, a class-C floral gene) in Arabidopsis [14, 28–30]. The activation of *AP3* expression requires the activity of both *LFY* and *UFO*, which likely explains the reported downregulation of class-B genes in the *ufo* mutant in Arabidopsis [13, 14] and class-C genes in the *apo1* mutant in rice [12]. In the developing spikes of the wheat *wapo1* null mutants, we also observed a highly significant downregulation of class-B, and -C genes relative to the WT, suggesting a conserved molecular mechanism. For the class-E genes we observed a highly significant downregulation of *SEP3-2* and only marginally significant differences for the three *SEP1* genes.”

The PLOS Publication Ethics team reviewed this case in collaboration with the *PLOS Genetics* Editors, and carefully considered case details including the confirmed data manipulation, that the data in question comprise a relatively minor portion of the results, and the reliability of the article’s main findings as demonstrated by the repeat data and supported by an Editorial Board member. PLOS issues this Expression of Concern to inform readers of the data manipulation concerns and that findings in Fig 3 and S1 Data published in [1] are unreliable. However, the journal stands by the article once amended with this notice to include these alerts and the data obtained in repeat experiments.

The Kronos near isogenic line with the H2 introgression was deposited in the National Small Grains Collection (PI 698810). All data are presented in the text and supplementary materials. The raw data for all figures and Supplemental Tables are available in S1 Data. The updated data “D” for S1E–S1G Fig is available in S1 File provided with this notice.

## Supporting information

S1 File S1 Data file with updated data for “D”.

S1 Fig Floral abnormalities and expression profiles of Kronos *wapo1* loss-of-function CRISPR mutant (*wapo-A1 wapo-B1*). Modified Fig 3 (E-G) Expression analysis of class-B, -C and -E MADS-box genes in Kronos and *wapo1* mutants relative to *ACTIN* (MADS-box gene nomenclature is based on [20]). Primers for the qRT-PCRs are listed in S1 Table. Bars represent averages of five replicates and error bars are s.e.m. Each replicate is a pool of six developing spikes at the early stamen primordia stage (W4.0 in the Waddington scale). ns = not significant, * = P < 0.05, ** = P < 0.01, and *** = P < 0.001. Data and statistical analyses used in E-G are available in the New Data D in S1 File.
(TIF)
