# Supplementary material for: WAPO-A1 is the causal gene of the 7AL QTL for spikelet number per spike in wheat
Source: PLoS Genet. 2022 Jan 13;18(1):e1009747. doi: 10.1371/journal.pgen.1009747 (PMC8791482; doi:10.1371/journal.pgen.1009747)
Supplement: S1 Table — (DOCX) [file pgen.1009747.s001.docx]

**S1 Table.** Primers used in this study

| **Function & name** | **Primer sequence** | **Comment** |
| --- | --- | --- |
| Guide RNA CRISPR F | ACTTGAGCGCCTCTCCCCCACCGT (Golden Gate cloning site)^a^ | Guide RNA for |
| Guide RNA CRISPR R | AAACACGGTGGGGGAGAGGCGCTC (Golden Gate cloning site)^a^ | CRISPR |
| g641-NGS-F | TCTACACGTTCAGAGTTCTACAGTCCGACGATCGGCCACCTCCTCCTCCTC | Screen CRISPR |
| g641-NGS-R | GTGACTGGAGTTCAGACGTGTGCTCTTCCGATCGTCGGCGACGAAGGTGTC | Mut. Next Gen. Seq. |
| CAPS-*WAPO-A1* F | ACTCTCACCTCCTCCACTCC | Marker for *WAPO-A1* |
| CAPS-*WAPO-A1* R1 | TCAAAGGACACAGATCAACC | mutation |
| CAPS-*WAPO-B1* F | ACTCTCACCTCCTCCACTCG | Marker for *WAPO-B1* |
| CAPS-*WAPO-B1* R2 | CATAATCAGAAATTTGCACAAGAAC | mutation |
| *WAPO1*-*Xho*I-F | CACCCTCGAGTCATTGGCTACAACAGTGGTCAG (restriction site) | Cloning genomic |
| *WAPO1*-*Avr*II-R | ATATCCTAGGAGATTTCTGGATGTTCCTTGTCA (restriction site) | *WAPO1* into pLC41 |
| *WAPO1*-LND-47F-F1 | CTGGCGTTCCTCCCGACGCCGTCCTT (mutation site) | Generation LDN-F47 |
| *WAPO1*-LND-47F-R1 | AAGGACGGCGTCGGGAGGAACGCCA (mutation site) | allele |
| *WAPO1*-Genotyping-F1 | TGACAAGGAACATCCAGAAATC | Genotyping transgenic |
| *WAPO1*-Genotyping-R2 | AGCCACCTTCCTTTTCCACT | genomic copy |
| qRTPCR-*WAPO-B1*-F1 | AGCTCAGCTCACTCACTCTCCC | qRT-PCR |
| qRTPCR-*WAPO-B1*-R1 | GGTGGTGAGGGTGTGGGTTC | *WAPO-B1* |
| *WAPO-A1*-RT-F2 ^b^ | CTCACTCACTCTCACTCCACG | qRT-PCR |
| *WAPO-A1*-RT-R2 ^b^ | GGTGGTGAGGCAGTAGGTTC | Tm*WAPO1* |
| qRTPCR-AP3-1-F  qRTPCR-AP3-1-R | AAAATGTCGATGCCGCTCTC  CTCCTGGGAGTGCTTCACCT | qRT-PCR *APETALA3* |
| qRTPCR-PI1-F  qRTPCR-PI1-R | AGATGCTGGAGGAGGAGCAC  CGGCATCTGGGAAGTGAAAT | qRT-PCR *PISTILLATA1* |
| qRTPCR-AG1-F  qRTPCR-AG1-R | AGACTGAAAGGGGGCAACAG  GCAGGAAGGTTCTCGGATCA | *qRT-PCR AGAMOUS1* |
| qRTPCR-AG2-F  qRTPCR-AG2-R | AACTTCCTGCAGGCGAACAT  TACTGCTGGCCGAGCTGAAG | *qRT-PCR AGAMOUS2* |
| qRTPCR-SEP1-2-F  qRTPCR-SEP1-2-R | GGAGCAAGAATTGCAGGATG  GCTASACTGCCCTCCGTCTT (S= 50% G + 50% C primer mix) | *qRT-PCR SEPALLATA1-2* |
| qRTPCR-SEP1-4-F  qRTPCR-SEP1-4-R | GGCGACAAAGAGCCAACAGT  TCCAACATCCTGGCAAGACA | *qRT-PCR SEPALLATA1-4* |
| qRTPCR-SEP1-6-F  qRTPCR-SEP1-6-R | GCAGCCAGAGCACTTCTTCC  GGCTGGTTCACATCCATGC | *qRT-PCR SEPALLATA1-6* |
| qRTPCR-SEP3-2-F  qRTPCR-SEP3-2-R | CCAACTTGCTCGGCTACGAC  TGCGTTGTTTATCTGCTCCTG | *qRT-PCR SEPALLATA3-2* |
| *ACTIN-F*  *ACTIN-R* | ACCTTCAGTTGCCCAGCAAT  CAGAGTCGAGCACAATACCAGTTG | *qRT-PCR ACTIN* |
| P3-WM-APO1-T3-F_1400_ | ATTAACCCTCACTAAAGGGAGCCATCGGCCTTCTGGAC (T3) | *WAPO1* *in situ* probe |
| P4-WM-APO1-T7-R_1649_ | TAATACGACTCACTATAGGGACTACCGATTCAAAACCTGT (T7) | *WAPO1* *in situ* probe |
| P5-WM-APO1-T7-R_1843_ | TAATACGACTCACTATAGGGGTTGGCTTGCCAAATATCTG (T7) | *WAPO1* *in situ* probe |

^a^ "G" is added as transcription start site for U6 promoter. ^b^ Primers for qRT-PCR of *WAPO-A1* are described in (1)

**Reference**

1. Kuzay S, Xu Y, Zhang J, Katz A, Pearce S, Su Z, et al. Identification of a candidate gene for a QTL for spikelet number per spike on wheat chromosome arm 7AL by high-resolution genetic mapping. Theor Appl Genet. 2019;132:2689–705. doi: 10.1007/s00122-019-03382-5. PMID: 31254024.
