# Supplementary material for: WAPO-A1 is the causal gene of the 7AL QTL for spikelet number per spike in wheat
Source: PLoS Genet. 2022 Jan 13;18(1):e1009747. doi: 10.1371/journal.pgen.1009747 (PMC8791482; doi:10.1371/journal.pgen.1009747)
Supplement: S2 Table — (DOCX) [file pgen.1009747.s002.docx]

**S2 Table.** F_2_ CRISPR loss-of-function mutants *WAPO-A1* x *WAPO-B1* factorial ANOVA for spikelet number per spike (SNS), heading date and leaf number of the main tiller at heading. Raw data and summary statistics are available in the Supplemental data file (Fig 1 and S2 Table spreadsheet).

**A.** Spikelet number per spike (supports Fig 1D)

Sum of Mean

Source DF Squares Square F Value Pr > F

Model 3 115.03 38.34 62.07 <.0001

Error 22 13.59 0.62

Corrected Total 25 128.62

R-Square = 0.89

Type III Mean

Source DF SS Square F Value Pr > F

*Wapo-A1* 1 23.33 23.33 37.78 <.0001

*Wapo-B1* 1 90.94 90.94 147.23 <.0001

*Wapo-A1*Wapo-B1* 1 4.97 4.97 8.04 0.0096

**Simple Effects**

Contrast DF Contrast Mean

SS Square F Value Pr > F Kruskal-Wallis

*Wapo-A1* in *wapo-B1* mut. 1 24.08 24.08 38.990 <.0001 0.0023

*Wapo-A1* in *Wapo-B1* WT 1 3.51 3.51 5.680 0.0263 0.0802

*Wapo-B1* in *wapo-A1* mut. 1 23.33 23.33 37.780 <.0001 0.0011

*Wapo-B1* in *Wapo-A1* WT 1 23.47 23.47 37.990 <.0001 0.0045

We could not find a transformation to restore normality of residuals in all the simple effects so we used non-parametric Kruskal-Wallis tests for the *P* values in the Figure 1D.

**B.** Days to heading. Not significant

Sum of Mean

Source DF Squares Square F Value Pr > F

Model 3 3.18 1.06 0.63 0.6020

Error 22 36.97 1.68

Corrected Total 25 40.15

R-Square = 0.07

Type III Mean

Source DF SS Square F Value Pr > F

*Wapo-A1* 1 0.21 0.21 0.12 0.7279

*Wapo-B1* 1 0.21 0.21 0.12 0.7279

*Wapo-A1*Wapo-B1* 1 2.90 2.90 1.72 0.2026

**C.** Leaf number on the main tiller. Not significant

Sum of Mean

Source DF Squares Square F Value Pr > F

Model 3 1.11 0.37 2.82 0.0625

Error 22 2.89 0.13

Corrected Total 25 4.00

R-Square 0.28

Type III Mean

Source DF SS Square F Value Pr > F

*Wapo-A1* 1 0.02 0.02 0.15 0.7062

*Wapo-B1* 1 0.48 0.48 3.65 0.0693

*Wapo-A1*Wapo-B1* 1 0.48 0.48 3.65 0.0693
