# Supplementary material for: WAPO-A1 is the causal gene of the 7AL QTL for spikelet number per spike in wheat
Source: PLoS Genet. 2022 Jan 13;18(1):e1009747. doi: 10.1371/journal.pgen.1009747 (PMC8791482; doi:10.1371/journal.pgen.1009747)
Supplement: S3 Table — (DOCX) [file pgen.1009747.s003.docx]

**S3 Table.** ANOVAs for 2020 and 2021 experiments testing the effect of H3 and H1 haplotypes on SNS, grain number per spike (GNS), grain yield, thousand kernel weight, and heading date. Split-plot RCBD with 10 replications, 8 families as main plots, and H1 and H3 haplotypes as sub-plots. Comparison between alleles within individual families for SNS were done using *t*-tests. These statistical analyses support Fig 7 in the main manuscript. Raw data and descriptive statistics are available in the Supplemental data file (Fig7A-D & S3 Table spreadsheet).

**A.** Spikelet number per spike (SNS). Field Experiment year 2020.

Sum of Mean

Source DF Squares Square F Value Pr > F

Model 83 424.16 5.11 10.58 <.0001

Error 59 28.49 0.48

Corrected Total 142 452.64

R-Square = 0.94

Type III Mean

Source DF SS Square F Value Pr > F

Block 9 23.91 2.66 5.50 <.0001

Family 7 301.34 43.05 101.57 <.0001

Block*Family 59 25.01 0.42 0.88 0.6909

**Allele (H3 vs H1)** 1 41.42 41.42 85.79 **<.0001**

Allele*Family 7 8.80 1.26 2.60 0.0207

Error 59 28.49 0.48

Level of -----SNS-------

Allele N Mean Std Dev

H3 72 22.21 1.72 6.0% increase in SNS

H1 71 20.95 1.63

ANOVAs by family.

Family H3 H1 *P*

12 21.2 20.1 *

19 21.9 21.7 ns

51 20.3 19.2 ***

55 23.0 21.5 **

69 21.0 19.6 ***

113 23.1 21.2 ***

120 25.3 23.9 **

128 21.0 20.2 ns

**B.** Spikelet number per spike (SNS). Field Experiment year 2021.

Sum of Mean

Source DF Squares Square F Value Pr > F

Model 86 508.12 5.91 34.94 <.0001

Error 69 11.67 0.17

Corrected Total 155 519.79

R-Square = 0.96

Type III Mean

Source DF SS Square F Value Pr > F

Block 9 11.03 1.23 7.24 <.0001

Family 7 338.27 48.32 188.98 <.0001

Block*Family 62 15.85 0.26 1.51 0.0476

**Allele (H3 vs H1)** 1 129.74 129.74 767.15 **<.0001**

Allele*Family 7 6.35 0.91 5.37 <.0001

Error 69 11.67 0.17

Level of -----SNS-------

Allele N Mean Std Dev

H3 79 24.03 1.68 8.2% increase in SNS

H1 77 22.21 1.50

ANOVAs by family

Family H3 H1 *P*

12 22.9 20.6 ***

19 24.2 22.9 ***

51 22.1 20.5 ***

55 24.2 22.0 ***

69 23.3 21.6 ***

113 24.6 22.7 ***

120 27.7 25.2 ***

128 23.0 21.6 ***

**C.** Grain number per spike (GNS). Field Experiment year 2021.

Sum of Mean

Source DF Squares Square F Value Pr > F

Model 86 6711.74 78.04 3.90 <.0001

Error 69 1382.37 20.03

Corrected Total 155 8094.12

R-Square = 0.98

Type III Mean

Source DF SS Square F Value Pr > F

Block 9 464.62 51.63 2.58 0.0128

Family 7 3924.02 560.57 17.77 <.0001

Block*Family 62 1956.01 31.55 1.57 0.0335

**Allele (H3 vs H1)** 1 169.31 169.31 8.45 **0.0049**

Allele*Family 7 127.35 18.19 0.91 0.5055

Error 69 1382.37 20.03

Level of ------GNS-------

Allele N Mean Std Dev

H3 79 70.33 7.31 3.29% increase

H1 77 68.09 7.01

**D.** Grain number per spikelet (spikelet fertility, GNS / SNS). Field Experiment year 2021.

Sum of Mean

Source DF Squares Square F Value Pr > F

Model 86 10.663 0.124 3.37 <.0001

Error 69 2.537 0.037

Corrected Total 155 13.200

R-Square = 0.81

Type III Mean

Source DF SS Square F Value Pr > F

Block 9 1.031 0.115 3.12 0.0033

Family 7 4.924 0.703 13.43 <.0001

Block*Family 62 3.247 0.052 1.42 0.0764

**Allele (H3 vs H1)** 1 0.873 0.873 23.73 **<.0001**

Allele*Family 7 0.268 0.038 1.04 0.4112

Error 69 2.537 0.037

Level of ----Fertility----

Allele N Mean Std Dev

H3 79 2.930 0.311 4.6% decrease in fertility

H1 77 3.071 0.254

**E.** Thousand kernel weight. Field Experiment year 2021.

Sum of Mean

Source DF Squares Square F Value Pr > F

Model 86 2939.66 34.18 3.77 <.0001

Error 69 626.43 9.08

Corrected Total 155 3566.09

R-Square = 0.82

Type III Mean

Source DF SS Square F Value Pr > F

Block 9 46.65 5.18 0.57 0.8163

Family 7 1850.87 264.41 23.07 <.0001

Block*Family 62 713.43 11.51 1.27 0.1687

**Allele (H3 vs H1)** 1 45.68 45.68 5.03 **0.0281**

Allele*Family 7 234.93 33.56 3.70 0.0019

Error 69 626.43 9.08

Level of ------TKW-------

Allele N Mean Std Dev

H3 79 61.70 4.83 1.56% decrease

H1 77 62.68 4.74

**F.** Total grain yield (kg/ha). Field Experiment year 2021.

Sum of Mean

Source DF Squares Square F Value Pr > F

Model 86 691451968 8040139 11.21 <.0001

Error 69 49485800 717185

Corrected Total 155 740937768

R-Square = 0.93

Type III Mean

Source DF SS Square F Value Pr > F

Block 9 26865041 2985004 4.16 0.0003

Family 7 536827378 76689625 68.46 <.0001

Block*Family 62 69449023 1120146 1.56 0.0360

**Allele (H3 vs H1)** 1 2745281 2745281 3.83 **0.0545**

Allele*Family 7 19273346 2753335 3.84 0.0014

Error 69 49485800 717185

Level of --------Yld--------

Allele N Mean Std Dev

H3 79 5798.83 2053.23 2.99% increase

H1 77 5630.73 2325.50

**G.** Heading time. Field Experiment year 2021.

Sum of Mean

Source DF Squares Square F Value Pr > F

Model 86 3467.99 40.33 35.81 <.0001

Error 69 77.71 1.13

Corrected Total 155 3545.69

R-Square = 0.98

Type III Mean

Source DF SS Square F Value Pr > F

Block 9 30.66 3.41 3.02 0.0042

Family 7 3316.87 473.84 285.01 <.0001

Block*Family 62 103.08 1.66 1.48 0.0579

**Allele (H3 vs H1)** 1 0.31 0.31 0.28 **0.5987**

Allele*Family 7 19.36 2.77 2.46 0.0262

Error 69 77.71 1.13

Level of ------HD--------

Allele N Mean Std Dev

H3 79 142.32 4.89 No significant difference

H1 77 142.14 4.70
