# Supplementary material for: WAPO-A1 is the causal gene of the 7AL QTL for spikelet number per spike in wheat
Source: PLoS Genet. 2022 Jan 13;18(1):e1009747. doi: 10.1371/journal.pgen.1009747 (PMC8791482; doi:10.1371/journal.pgen.1009747)
Supplement: S4 Table — (DOCX) [file pgen.1009747.s004.docx]

**S4 Table.** ANOVAs for 2021 experiment to evaluate the effect of *WAPO-A1* haplotype H2 introgressed into tetraploid Kronos (H1 haplotype, CRD n = 9 per genotype) and hexaploid high-biomass line GID4314513 (H1 haplotype, RCBD n = 10 blocks). These statistical analyses support Fig 8 in the main manuscript. Raw data and descriptive statistics are available in the Supplemental data file (Fig 8 & S4 Table spreadsheet).

**Tetraploid wheat Kronos (A-F)**

**A.** Spikelet number per spike (SNS).

Sum of Mean

Source DF Squares Square F Value Pr > F

Allele 1 58.32 58.32 186.62 <.0001

Error 16 5.00 0.31

Corrected Total 17 63.32

R-Square: 0.9210

Level of ---------SNS--------

Allele N Mean Std Dev

H1 9 20.367 0.702

H2 9 23.967 0.364

**B.** Grain number per spike (GNS)

Sum of Mean

Source DF Squares Square F Value Pr > F

Allele 1 3.4 E-9 3.4 E-9 6.08 0.0253 *

Error 16 8.9 E-9 5.5 E-10

Corrected Total 17 1.2 E-8

R-Square: 0.275426. Data transformed to the Power -2 to restore normality.

Level of --------GNS---------

Allele N Mean Std Dev

H1 9 70.122 5.860

H2 9 74.800 2.789

**C.** Spikelet fertility (grain number per spikelet = GNS / SNS)

Sum of Mean

Source DF Squares Square F Value Pr > F

Allele 1 0.4557 0.4557 19.62 0.0004

Error 16 0.3715 0.0232

Corrected Total 17 0.8272

R-Square: 0.275426.

Level of ------SpkFert------

Allele N Mean Std Dev

H1 9 3.439 0.186

H2 9 3.121 0.108

**D.** Grain weight per spike.

Sum of Mean

Source DF Squares Square F Value Pr > F

Allele 1 0.41 0.41 2.90 0.1078

Error 16 2.25 0.14

Corrected Total 17 2.66

R-Square: 0.1536

Level of -----SpkGrWgt------

Allele N Mean Std Dev

H1 9 4.239 0.480

H2 9 4.540 0.226

**E.** Thousand Kernel Weight (TKW).

Sum of Mean

Source DF Squares Square F Value Pr > F

Allele 1 0.231 0.231 0.01 0.9140

Error 16 307.155 19.197

Corrected Total 17 307.387

R-Square: 0.00075

Level of --------TKW--------

Allele N Mean Std Dev

H1 9 60.503 5.524

H2 9 60.730 2.806

**F.** Days to Heading (DTH).

Sum of Mean

Source DF Squares Square F Value Pr > F

Allele 1 4.50 4.50 2.12 0.1649

Error 16 34.00 2.12

Corrected Total 17 38.50

R-Square: 0.116883

Level of ---------DTH---------

Allele N Mean Std Dev

H1 9 136.333 1.323

H2 9 137.333 1.581

**High biomass hexaploid line GID4314513**.

**G.** GID4314513. Spikelet number per spike (SNS).

Sum of Mean

Source DF Squares Square F Value Pr > F

Model 10 11.97 1.20 1.60 0.2449

Error 9 6.72 0.75

Corrected Total 19 18.68

R-Square: 0.6406

Source DF Type III Mean F Value Pr > F

SS Square

Block 9 3.84 0.43 0.57 0.7911

Genotype 1 8.13 8.13 10.89 0.0092

Mean H2 = 22.13 ± 0.14, H1 = 20.85 ± 0.31

**H.** GID4314513. Grain yield (kg/ha).

Sum of Mean

Source DF Squares Square F Value Pr > F

Model 10 5561338 556134 2.80 0.0687

Error 9 1790642 198960

Corrected Total 19 7351980

R-Square: 0.7564

Source DF Type III Mean F Value Pr > F

SS Square

Block 9 4539438 504382 2.54 0.0910

Genotype 1 1021900 1021900 5.14 0.0497

Mean H2 = 5860.9 ± 220.5, H1 = 5408.9 ± 147.3

**I.** GID4314513. Grain number per spike (GNS).

Sum of Mean

Source DF Squares Square F Value Pr > F

Model 10 168.50 16.85 0.51 0.8468

Error 9 298.42 33.16

Corrected Total 19 466.92

R-Square: 0.360872

Source DF Type III Mean F Value Pr > F

SS Square

Block 9 152.30 16.92 0.51 0.8346

Genotype 1 16.20 16.20 0.49 0.5022

Mean H2 = 69.5 ± 1.7, H1 = 67.7 ± 1.4.

**J.** GID4314513. Spikelet fertility (grains per spikelet = GNS / SNS).

Sum of Mean

Source DF Squares Square F Value Pr > F

Model 10 0.35 0.035 1.04 0.4795

Error 9 0.30 0.033

Corrected Total 19 0.65

R-Square: 0.536779

Source DF Type III Mean F Value Pr > F

SS Square

Block 9 0.29 0.033 0.97 0.5179

Genotype 1 0.06 0.057 1.70 0.2245

Mean H2 = 3.1 ± 0.07, H1 = 3.2 ± 0.05.

**K.** GID4314513. Grain weight.

Sum of Mean

Source DF Squares Square F Value Pr > F

Model 10 49.78 4.98 1.94 0.1662

Error 9 23.07 2.56

Corrected Total 19 72.84

R-Square: 0.683339

Source DF Type III Mean F Value Pr > F

SS Square

Block 9 44.93 4.99 1.95 0.1675

Genotype 1 4.85 4.85 1.89 0.2022

Mean H2 = 56.9 ± 0.6, H1 = 55.9 ± 0.3.

**L.** GID4314513. Grain weight per spike.

Sum of Mean

Source DF Squares Square F Value Pr > F

Model 10 0.653 0.065 0.67 0.7273

Error 9 0.873 0.097

Corrected Total 19 1.527

R-Square: 0.427963

Source DF Type III Mean F Value Pr > F

SS Square

Block 9 0.509 0.057 0.58 0.7833

Genotype 1 0.144 0.144 1.49 0.2534

Mean H2 = 3.96 ± 0.10, H1 = 3.79 ± 0.08.
