# Supplementary material for: WAPO-A1 is the causal gene of the 7AL QTL for spikelet number per spike in wheat
Source: PLoS Genet. 2022 Jan 13;18(1):e1009747. doi: 10.1371/journal.pgen.1009747 (PMC8791482; doi:10.1371/journal.pgen.1009747)

**S1 Figure**. Expression analysis of *WAPO-A1* and *WAPO-B1* homeologs in developing spikes. (**A**) Quant-Seq data from Kronos (Li et al. The Plant Cell 2021 doi:10.1093/plcell/koab243). (**B**) Unpublished RNAseq from our lab at the same spike development stages in Kronos. VEG = vegetative stage, DR = double ridge, PDR = post-double ridge, and TS = terminal spikelet stage. TPM= transcripts per million. (**C**) qRT-PCR analysis of *WAPO1* expression in Kronos developing spikes at the terminal spikelet stage (TS). Values are ΔCt using *ACTIN* as endogenous control. Averages are based on four biological replications per stage in each experiments and error bars are s.e.m. ns = not significant, * = *P* < 0.05, ** = *P* < 0.01, and *** = *P* < 0.001.


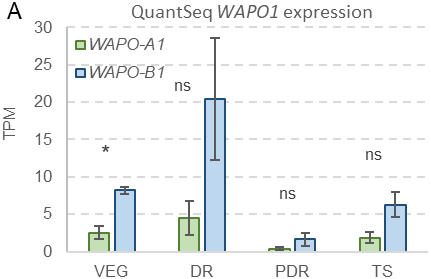

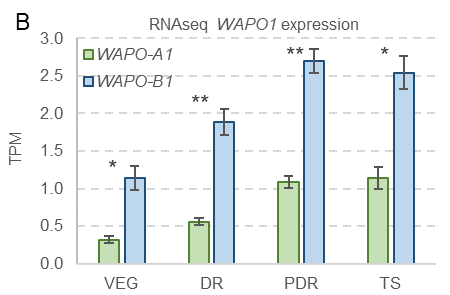

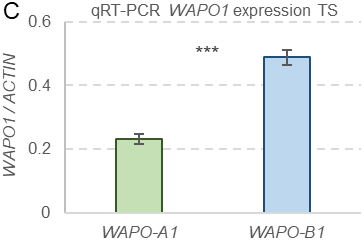

Supplement: S1 Fig — (DOCX) [file pgen.1009747.s005.docx]
