# Supplementary material for: WAPO-A1 is the causal gene of the 7AL QTL for spikelet number per spike in wheat
Source: PLoS Genet. 2022 Jan 13;18(1):e1009747. doi: 10.1371/journal.pgen.1009747 (PMC8791482; doi:10.1371/journal.pgen.1009747)
Supplement: S2 Fig — (DOCX) [file pgen.1009747.s006.docx]

**S2 Figure.** Strongest floral abnormalities in Kronos plants transformed with *WAPO-A1* genomic regions of LDN-F47-2. (**A**) Spikes of a transgenic plant with the spikelets removed to show the distribution of naked pistils. (**B**) Detail of the naked pistils, showing the gradual transition to bracts when moving to more apical spikelet positions. (**C**) Gradation of the changes in spikelet and floret morphology from basal spikelets (1) to the terminal spikelets. Only spikelets from one side of the spike are shown (uneven numbers). (**D-G**) Basal spikelets of WT and LDN-F47-4 showing differences in organ size. (**D**) Glumes. (**E**) Fourth floret of the basal spike. (**F** and **G**) Lodicules, anthers and pistils in the third and fourth floret, respectively. Lm = lemma, Pa = palea, Ld = lodicule, An = Anther, Pi = pistil, ov = ovary.

**
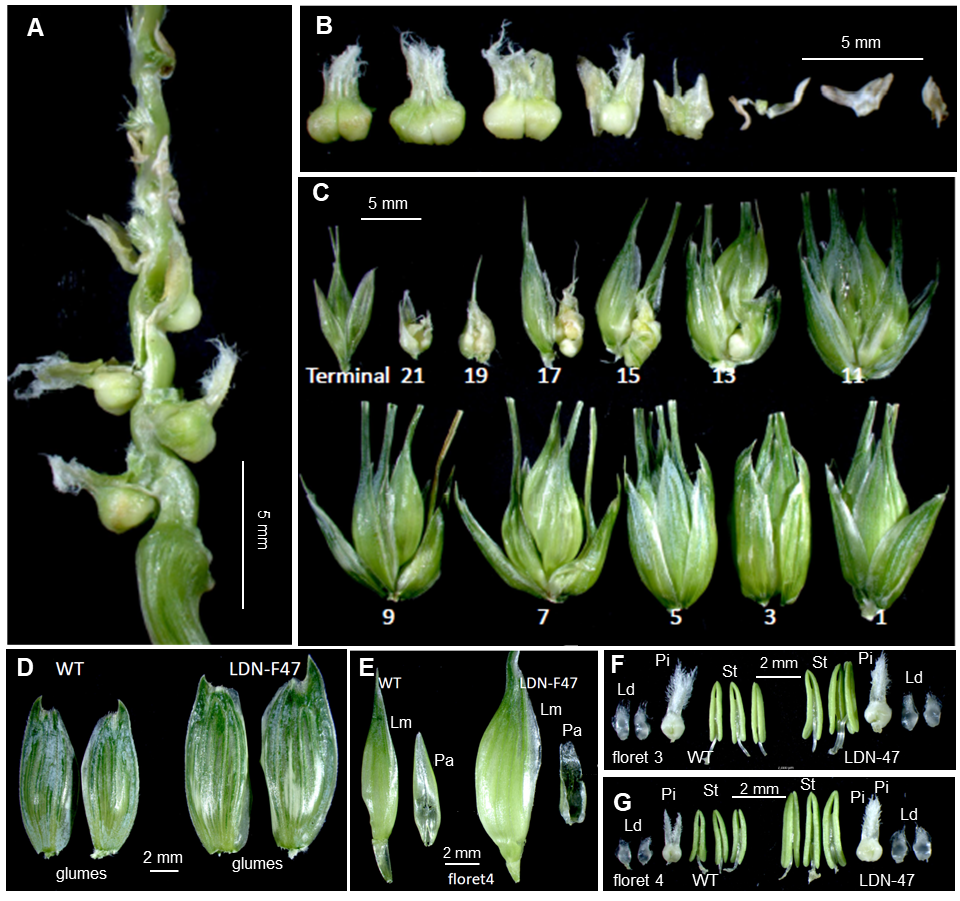
**
