# Supplementary material for: WAPO-A1 is the causal gene of the 7AL QTL for spikelet number per spike in wheat
Source: PLoS Genet. 2022 Jan 13;18(1):e1009747. doi: 10.1371/journal.pgen.1009747 (PMC8791482; doi:10.1371/journal.pgen.1009747)
Supplement: S3 Fig — (DOCX) [file pgen.1009747.s007.docx]

**S3 Figure.** *In situ* hybridization of *WAPO1* probe amplified from *T. monococcum* in developing spikes of diploid wheat *T. monococcum.* (**A**-**C**) Antisense *WAPO1* probe. (**D**) Sense *WAPO1* probe used as a negative control in a developing spike with undifferentiated spikelet meristems (SM) (W2.5). (**A**) Developing spike at the double ridge stage (W2.5), showing weak expression of *WAPO1* in the inflorescence meristem (IM). (**B**) Early spike development with undifferentiated spikelet meristems (SM) (W3). Weak *WAPO1* expression was detected in the IM and spikelet meristem (SM). (**C**) More advanced spike development (W3.25-3.5) showing strong expression of *WAPO1* in floret meristems (FM) in the more advanced central spikelets. W values indicate developmental stages based on the Waddington scale. Red arrows indicate regions where *WAPO1* hybridization signal was detected.

**
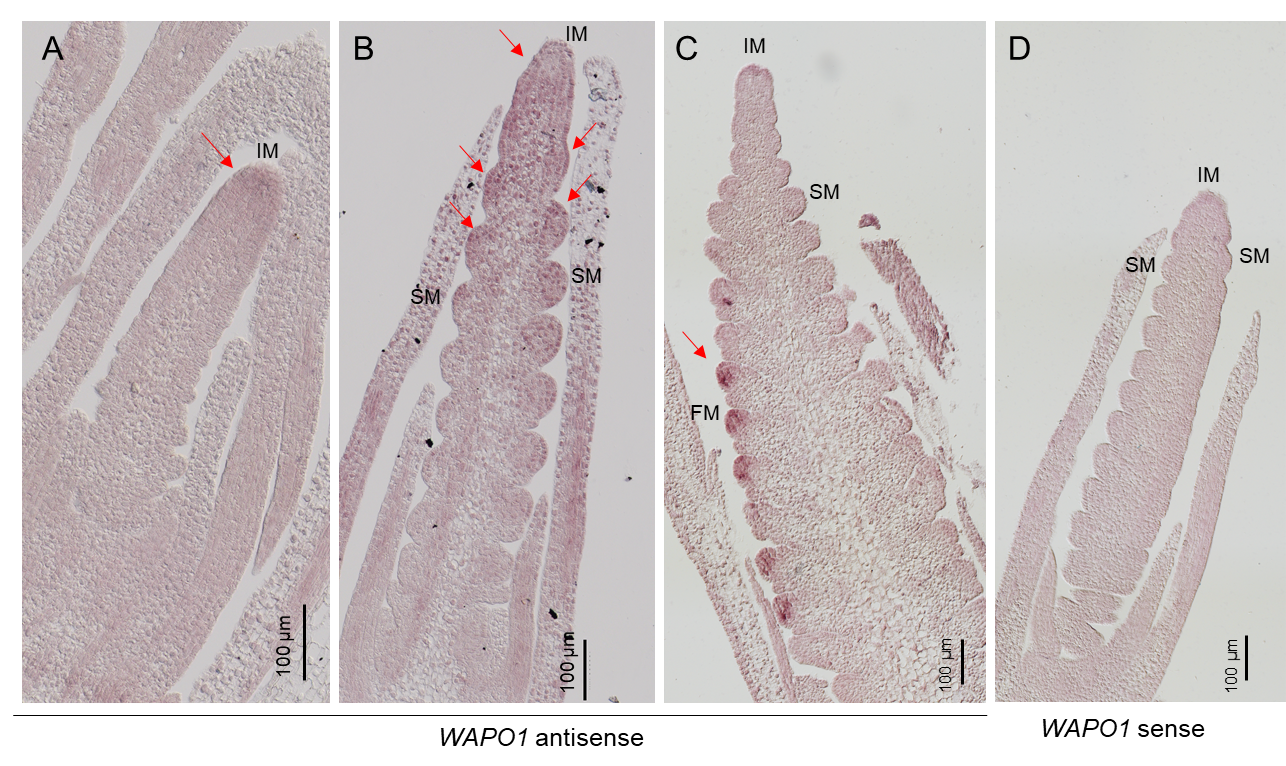
**
