# Supplementary material for: WAPO-A1 is the causal gene of the 7AL QTL for spikelet number per spike in wheat
Source: PLoS Genet. 2022 Jan 13;18(1):e1009747. doi: 10.1371/journal.pgen.1009747 (PMC8791482; doi:10.1371/journal.pgen.1009747)
Supplement: Attachment — Submitted filename: Response to reviewers.pdf [file pgen.1009747.s009.pdf]

Dear Editor

Thank you for the suggestions and corrections. Below is a list of the changes that we introduced in the manuscript to address them. The changes are also indicated with track changes in the revised manuscript. In blue are sections changed in the manuscript.

Editor comments

1. I would have liked SEMs or in situs but realize that they may or may not be informative.

Answer: We expanded our research team to bring the necessary expertise to add the requested experiments. We added *in situ* hybridization analyses of *WAPO1* at three spike developmental stages in both tetraploid wheat Kronos and diploid wheat *T. monococcum.* We added a new Results section to describe the *in situ* results that include a new Figure 6 with the *in situs* for Kronos and S3 Fig for the *in situs* for *T. monococcum.* Expression of *WAPO1* was detected in the inflorescence and spikelet meristems in the early stages of spike development and in the floret meristem in the later stages. This expression profile agrees with the effects of this gene on spike architecture and flower development presented in the paper. A comparison of the *WAPO1 in situ* profiles in wheat and those recently published in barley is included in the discussion.

2. I agree that it would be good to know if the MYC tagged protein is stable. MYC antibodies are available and this should not be a difficult experiment. Be sure to use inflorescences of the appropriate stage.

Answer: We followed the suggestion from Reviewer #1 to eliminate the transgenic plants expressing *WAPO1* under the maize *UBI* promoter, so this question is no longer relevant. See our justification in that answer to the second question from Reviewer #1.

3. *I believe Kronos has the H1 haplotype from Table 1. perhaps make that obvious at first mention of Kronos*

Answer: In the first paragraph of Material and Methods we added: "Kronos has the *WAPO-A1* H1 haplotype, which carries a 115-bp deletion in the promoter and a change from aspartic acid to asparagine at position 384 (D384N)."

In the Results section, we added: "A search of the sequenced EMS-mutagenized population of tetraploid wheat Kronos (Krasileva et al. 2017), which carries the *WAPO-A1* H1 haplotype, yielded one mutant line with a premature stop codon in *WAPO-A1* but no stop codon or splicing site mutations were detected in the *WAPO-B1* homeolog."

4. line 208-. Mention is made of transcript levels for one line. Are the data for all the lines in the supplemental figure? Is this worth showing in the regular figures?

Answer: We added transcript analyses of *WAPO-A1* in transgenic plants transformed with three different constructs. For each transgenic construct, we analyzed *WAPO-A1* transcript levels in

one sister line without the transgene (WT), one with weak spike and floral phenotypes and one with strong phenotypes. The results, described in new Figure 5 and Supplemental data, show that the transgenic plants for all three constructs have significantly higher *WAPO-A1* transcript levels than their corresponding wild type sister lines. In all three constructs the progeny of the plants with the strongest spike and floral phenotype showed the highest *WAPO1* transcript levels.

5. The figures of the floral organs need a wild-type panel.

Answer: We included a wild-type panel in Figure 4C to show a normal Kronos floret. We also expanded the panels in Figures 3 and 4 to include paleae and lemmas. These organs are normal in the *WAPO1* mutants.

6. Do these abnormalities interfere with fertility? I know the SNS number increases but are the florets all fertile?

Answer: We estimated spike fertility as the average number of grains per spikelet (GNS /SNS) and incorporated these results in the new Figure 5D-F. We also determined the average number of florets per spikelet. We added the new statistical data in the Supplemental data file under the spreadsheet Fig 5. We added in the Results section: "In all three experiments, the progeny of plants from events with strong spike phenotypes (naked pistils and small terminal spikelet) showed higher WAPO1 transcript levels (Fig 5A-C), and stronger reductions in number of florets per spikelet (Fig.5D-F) and average spikelet fertility (Fig 5G-I) than the progeny of the events with weaker spike phenotypes and intermediate *WAPO-A1* expression levels. The progeny of F47-2-1 segregated for weak and strong spike phenotypes and the number of florets per spikelet and spikelet fertility was more affected than in the progenies of the other two constructs with intermediate levels of *WAPO-A1* expression."

We added a comment in the discussion indicating that: "In addition, mutants and transgenic plants with strong floral abnormalities showed reduced fertility".

We are not advocating the use of these *WAPO1* transgenic plants for breeding. The main objectives of generating these transgenic plants were for candidate gene validation and functional characterization. For breeding purposes, we propose the use of the natural mutation in haplotype 2. Haplotype H2 was associated with significant increases in both SNS and grain number per spike in field experiments, suggesting limited or non-existent negative effects on fertility.

7. line 275.observed in some plants. Please give frequency

Answer: Frequencies were added: "This abnormality was observed in transgenic plants of events TmDV92-1 (in 4 out of 6 T1), LDN-C47-3 (in 3 out of 9 T1), LDN-F47-2 (in 8 out of 21 T1) and LDN-F47-6 (in 3 out of 18 T1) (Fig 4A-B and S2 Fig). Transgenic line LDN-F47-2 had the most extreme phenotype showing multiple naked pistils, which were larger at the basal spikelets and transitioned to bracts in the more distal spikelets (S2A-B Fig)."

8. line 320. end of sentence is missing

Answer: Sorry, the word "frequent" was missing at the end. This sentence was deleted from the revised paper since we eliminated all the results related to the *UBI::WAPO1:MYC* transgenic plants.

9. please be consistent with colors in the figures (I think this was mentioned also by reviewer 1).

Answer: We used blue for the WT in Figures 1, 2 and new figure 5. In new Figure 7 we used blue for the H3 haplotype, which is the ancestral haplotype, and equivalent to a wild type. As requested by the reviewer, we also moved the WT to the left in all figures. In the last figure (new Figure 8), we changed H1 to orange to be consistent with Figure 7, and changed H2 to green.

10. line 490. trade off that (not trade off hat).

Answer: Corrected as suggested.

Reviewer #1

On the whole, the work provides solid genetic proof that the gene controls spikelet number in wheat. I do however have a number of concerns that should be addressed by the authors.

MAJOR COMMENTS

1) There are examples throughout the manuscript where the authors have presented statements about quantitative differences between genotypes but have not supported them with data in the figures or supplementary information. Can the authors please provide graphs or tables support this information because it is difficult to assess variation or the replication of this data. Also, it is not clear why the presented percentages do not include an indication of variation in the data (for example, mean ± SEM). This comment also relates to examples where the authors have declared a correlation between traits, but not shown the phenotypic scores to support the associations.

There are examples throughout the manuscript (e.g. Figure 3A-D, Figure 4) where the authors have provided statements about percentage differences between genotypes that have not been supported by quantitative data within the figure panels, or the supplementary figures. The authors should present quantitative data to support these statements

Answer: We generated a Supplemental data Excel file that includes all the raw data and descriptive statistics for all the figures, table and supplemental tables presented in the paper, each described in separate spreadsheets within the same file. The regressions between SNS and DTH are presented in the first spreadsheet. Regarding the percent of spikes and flowers showing particular defects in the *wapo1* mutants, we indicated the total number of observations that were used to calculate these percentages: "To establish the frequency of the different floral abnormalities, we dissected 91 florets from 32 spikelets located at different positions along the spike from 14 different *wapo1* mutant plants (Supplemental data file, spreadsheet Fig 3A-D)".

We added in the Supplemental data file spreadsheet “Fig 3A-D” a detailed description of the floral abnormalities found at different positions of the spikes for the 14 plants analyzed.

2) In relation to the lines that overexpress the MYC-tagged version of WAPO, do the authors have any data to suggest that a functional protein is being produced in these lines? It is inconsistent with the transgenic lines that express LDN-F47 and LDN-C47 (Figure 2) that the UBI lines do not show a significant difference in spikelet number, and it indicates that the proteins may not be functional. Also, while I appreciate that they show higher expression in leaves, this does not necessarily mean the gene is overexpressed in the developing inflorescences. Is there any data showing higher expression in the developing inflorescences? Given the statement on L315, the authors have obviously considered the potential of the MYC protein interfering with the function of the protein, but they have not tested activity. I suggest the authors consider whether they need to include the data on the UBI overexpressing lines – does it add anything given the results of the LDN/TmDV92 transgenic lines, which are independent events?

Answer: We agree with the suggestion from Reviewer #1 to eliminate the results from the *UBI* overexpressing lines. We think that the paper reads better without these lines and that no relevant biological information is lost by excluding them. With three independent sets of transgenic lines using the natural *WAPO1* promoter, the addition of the *UBI* lines is redundant and more difficult to interpret than the results from the lines using the natural promoter. In the *UBI::WAPO1:MYC* experiments, *WAPO1* is expressed in tissues and developmental stages where it is not normally expressed and the biological relevance of the result is not clear. In addition, the effect of the MYC tag on the protein activity and interactions is not known. We thank Reviewer #1 for this suggestion.

3) Following on from this point, I am concerned about the comparison between transgenic lines on L322-326, and whether the phenotypes really are similar enough to conclude that the UBI and the genomic constructs both express active proteins. Firstly, it’s not clear based on the text used here whether the authors are trying to say that there were no significant phenotypic differences within plants of the same transgenic event, or among plants from the different transgenic events, or both? In the current version, I read it to mean that there are no significant phenotypic differences within plants of different transgenic events – if so, then I do not agree with their conclusion. For example, they do not share an effect on spikelet number, and the differences in heading date are difficult to examine because there is a 20-day difference in flowering time between the controls used for the LDN/TmDV92 transgenic lines and the UBI transgenic lines – why is this so, are they grown under different conditions/photoperiods? I agree that there are similar differences in floral architecture traits. I would like to ask whether the authors have considered the effect of the differences in higher expression of these two sets of transgenic lines – for example, if I interpret the graphs correctly, the UBI transgenic lines express WAPO >100-fold relative to wild-type, while the genomic constructs express the gene 8-10 fold higher (??) What affect would this difference have on the phenotypes? Again, I would like to ask the authors if they consider the genomic transgenic lines to be more insightful than the UBI transgenic lines, and whether the UBI transgenic lines should be included in the manuscript?

Answer: As indicated in our previous answer, we eliminated all the results related to the *UBI::WAPO1:MYC*, so the comparison with the transgenic lines with the natural promoter is no longer relevant. The point we were trying to make in the comparison between the *UBI::WAPO1:MYC* and the transgenic plants with the natural promoter, was that the addition of the MYC tag did not abolish the effect of the encoded protein since some of the phenotypes observed in the *UBI::WAPO1:MYC* plants resembled those of the transgenic plants with the natural promoter. We recognize that this was not well explained.

Regarding the possibility of comparing the lines with the three different natural promoters, the number of independent transgenic events for each construct was too small and the variability within event too large to make any valid comparisons. In addition, the differences among constructs were relatively small: the average SNS increases varied from 13.4 to 15.5% and the delays in heading time from 5.4 to 7%.

4) L286-288 – the authors should be careful with their conclusion about the association of more spikelets and the production of smaller distal spikelets in the WAPO1 transgenic lines. This is because the determination of spikelet number is temporally separate from the decision of a spikelet to form fertile florets, and without analysis of the developing inflorescences from the double ridge stage until emergence of the inflorescence, this conclusion may be too strong. For example, the spikelets of the transgenic lines may not be any different from the control lines during early stages, and the fertility of florets within the distal spikelets would be determined much later. There are also other reasons why the florets within distal spikelets could be infertile, including the floral architecture phenotypes shown be the authors in these lines – together, I recommend the authors reconsider this conclusion/statement, or ask that they perform further experiments to support it.

Answer: We eliminated this correlation and the associated conclusion, since it does not add any critical information for the paper.

5) L368-371 – In my opinion, the statistical analysis performed across the two developmental stages is unnecessary. It is perfectly reasonable to expect that WAPO could show more difference in expression at one particular stage (e.g. W3.5), but not all (W3.25), and the separation of the two stages tells us something important about the gene function that is lost by attempting to combine the stages – for example, this result suggests that the gene is being suppressed in H1 lines as inflorescence development progresses, but not in the H3 lines. Potentially the effect of the haplotypes occurs during later stages.

Answer: We agree with the reviewer that the experiments at the different developmental stages are more informative than the combined analysis. We eliminated the combined statistical analysis across the two developmental stages.

6) L462 – it is not true that paired spikelets form more frequently at the base of the spike: the Ppd-1 publication shows that they form predominantly within the central region.

Answer: Our mistake. We corrected the sentence to : "The naked pistils in the transgenic wheat plants appeared adjacent to and directly below normal spikelets, in a position similar to the paired spikelets observed in loss-of-function mutants for *ppd-D1* and *ft-B1* in hexaploid wheat

[33]. However, the paired spikelets in the previous two mutants were more frequent in the central part of the spike, whereas in the *WAPO-A1* transgenic plants the naked pistils were more frequent in the basal region of the spike."

7) L506 – in relation to the conclusion about not "significantly affecting heading time", the authors should clarify this statement by including "under field conditions", as experiments with the transgenic lines showed delayed heading time under glasshouse conditions.

Answer: We agree. We rephrased to: "In summary, the validation of *WAPO-A1* as the causal gene of the 7AL SNS QTL and of the positive effects of the H2 haplotype on spikelet and grain number per spike, provides wheat breeders a new tool to improve sink traits with limited effects on heading time."

MINOR COMMENTS

1) L107 - Can the authors check the names of the ancestral alleles and correct the names if they agree that it is not necessary to include "1" after Wapo, here.

Answer: The name of the wheat gene is *WHEAT ORTHOLOG OF APO1*, where *APO1* is the rice gene that corresponds to Arabidopsis *UFO*. The name of the gene in rice is *APO1* and there is a second gene in rice called *APO2*, therefore the presence of the number 1 is critical to indicate to which gene of rice we are referring. This name, was proposed in agreement between the authors of the previous QTL papers after extensive discussion and has been accepted as the official name of the gene by the Catalog of Gene Symbols for Wheat, which will make it difficult to change.

2) L131 – can the authors include detail in this sentence about how many amino acids are in the WAPO1 protein to support the statement about the mutant protein being truncated by 51%?

Answer: We added this information in the first paragraph of Results: "*WAPO-A1* mutation W216* identified in Kronos mutant line K4222 results in a premature stop codon and the truncation of 51 % of the encoded protein, which has 440 amino acids."

3) L145 – the use of the term "demonstrate" seems too strong here given the authors only examined one mutant here that could be influenced by associated background mutations. I understand the further work to come in the ms can support use of the term "demonstrate", but here I recommend they use "indicate" or "suggest".

Answer: We agree that it is premature to use the word "demonstrate" at this early section of the paper, before we present the CRISPR and transgenic results. We replaced "demonstrate" by "indicate" as suggested by the reviewer: "Taken together, these three experiments indicate that loss-of-function of the A-genome homeolog of *WAPO1* is sufficient to significantly reduce SNS."

4) For Figure 2, how many spikes were scored to generate this data? Can the authors include detail in the figure legend.

Answer: We recorded SNS only in the main spike of each transgenic plant. The number of plants measured for each event is indicated within each column. This information was added to the legend as requested.

5) As a general comment, the order of data should be corrected in the graphs to have the wild-type genotype on the left and the mutant or transgenic lines on the right. For example, in Figure 1A-C, WT should be on the left followed by wapo-A1. Also consider this idea for Figures 5 and 6 in reference to the haplotypes.

Answer: We changed Figures 1A-C to have the WT on the left. WT is on the left in all figures. In the haplotype comparisons, we put the ancestral H3 haplotype on the left (new Figure 7), because it is analogous to the WT. We also used the same light blue color for the WT and H3 ancestral haplotype in all graphs. In Figure 8, there is no WT or ancestral allele, but we kept the H1 haplotype on the right and use the same color as in Figure 7 for consistency.

6) Why are images within Figures 3 and 4 cut off in the figure panels? Are they of organs that do not show traits?

Answer: Since no phenotypic differences were observed in glumes of the wild type, the *wapo1*-null or the transgenic lines, we cut off the glume portions in those figures. We have now expanded the panels in these two figures to show the paleas and lemmas and part of the glumes when possible.

7) For Figure 5E-F, I recommend the authors describe the difference in expression in terms of "X-fold" rather than percentage difference.

Answer: We changed as suggested: "The first two experiments at W3.25, which were analyzed together using experiment as block, showed 1.8-fold higher transcript levels in H3 than in H1, but the difference was marginally not significant ($P = 0.07$, Fig 7E, Supplemental data). In the third experiment at W3.5, transcript levels in H3 were 3.0-fold higher than in H1 and the differences were highly significant ($P = 0.0003$, Fig 7F, Supplemental data)."

8) For Figure 5, I found it confusing that the x-axis labels include Kronos and Rusty – I recommend the authors use H1 and H3 with the designated names of the alleles in brackets (also for Figure 6 for H1 and H2). The use of the alleles would better reflect the HIFs that were used to test the effect of these haplotypes/alleles – in its current state, the names indicate that it the analyses were performed using Kronos and Rusty rather than the more informative germplasm that the authors generated.

Answer: Agree. In the new Figure 7, we eliminated the names of Kronos and Rusty from the x-axis labels and kept only the names of the alleles as suggested by the reviewer. We also eliminated the names of the parental lines from the legend. In the new Figure 8, we only have the haplotype designations in the x-axis and we eliminated the names Kronos and GID4314513 from

the titles. However, in the case the name of the recurrent parents in the legend is important since we are working in two different genetic backgrounds. It is also important to indicate which is the *WAPO-A1* allele present in each of these recurrent parents.

Reviewer #2

Albeit majorly confirmatory (in relation to previous works in At and rice!), this study is highly appreciated for wheat researchers and breeders, since it showcases what's required for functional gene analyses. I only have a few points for improvement:

1) The literature for the WAPO1 gene has not been properly covered!! The gene was initially discovered independently by three groups in parallel. Therefore, the paper by Muqaddasi et al. (2019) [TaAPO-A1, an ortholog of rice ABERRANT PANICLE ORGANIZATION 1, is associated with total spikelet number per spike in elite European hexaploid winter wheat (Triticum aestivum L.) varieties. Scientific Reports 9:13853; doi.org/10.1038/s41598-019-50331-9] must be added! Please include in line 80, line 92 and for the discovery of the F-box domain substitution C47F.

Answer: We apologize for the unintended omission. We added the Muqaddasi et al. (2019) reference in in the three places requested by Reviewer #2. New reference [7].

2) The claim in line 71 that grain yield has low heritability: I think this general statement is simply not true. GY has often heritabilities around 0.5, which is relatively high for a complex trait. Heritabilities for GY are often low in low-yielding ENVs with strong GxE effects. But in high-yielding ENVs it is often the opposite. I therefore suggest the authors to rather say: "Identifying genes controlling total GY is challenging due to its complex quantitative nature and GxE interactions." Please replace and update the citations #3 and #4.

Answer: We changed the sentence as suggested and updated the reference: "Identifying genes controlling total grain yield is challenging due to its complex quantitative nature and genotype by environment interactions [3]."

3) The correct plural form of 'palea' is 'paleae'; please change accordingly

Answer: We changed "paleas" to "paleae" in the five places where the plural was used
