# Supplementary figures and images for: Expression of Concern: WAPO-A1 is the causal gene of the 7AL QTL for spikelet number per spike in wheat
Source: PLoS Genet. 2023 Dec 20;19(12):e1011096. doi: 10.1371/journal.pgen.1011096 (PMC10732405; doi:10.1371/journal.pgen.1011096)

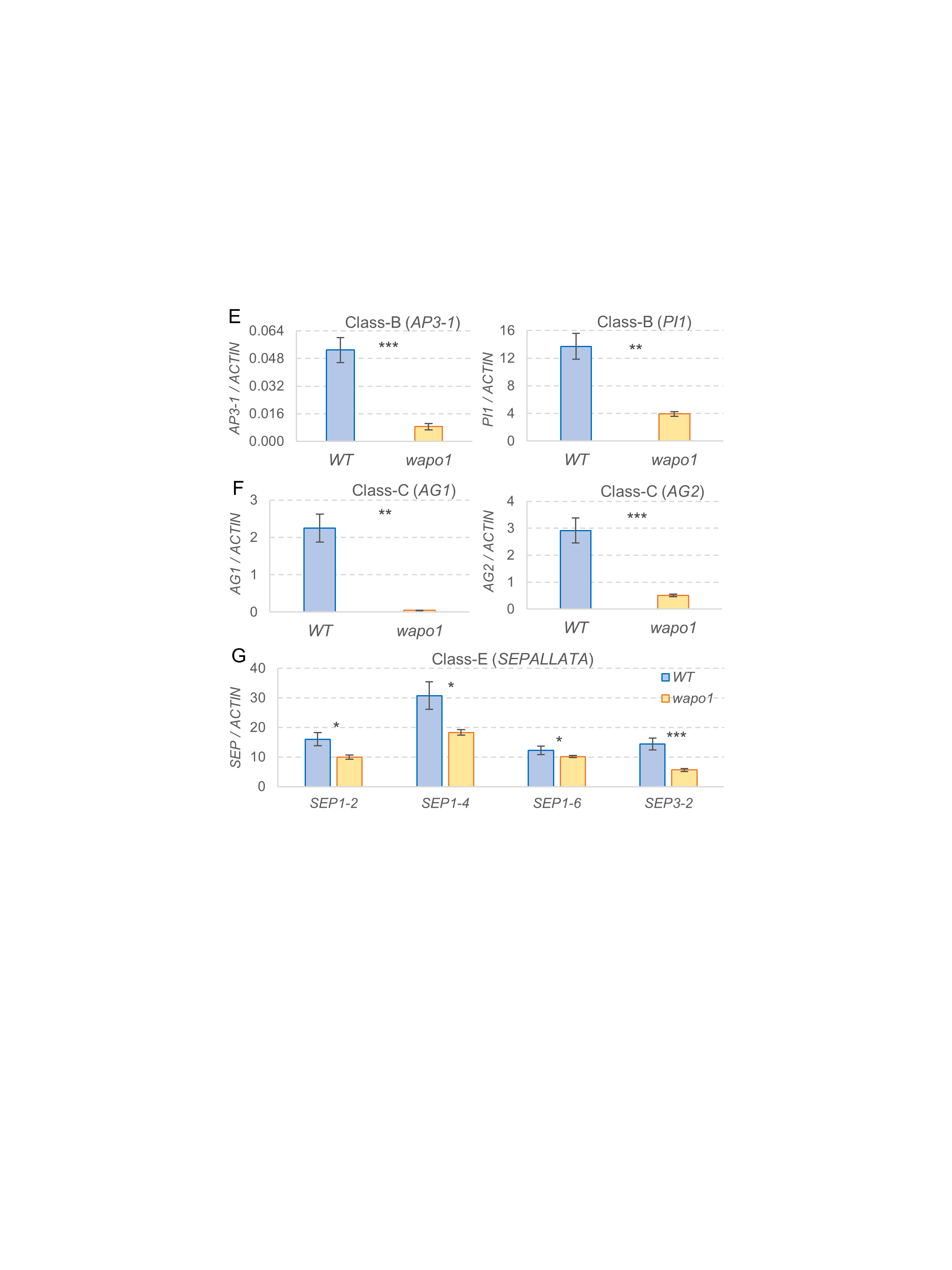

Supplement: S1 Fig — Modified Fig 3 (E-G) Expression analysis of class-B, -C and -E MADS-box genes in Kronos and wapo1 mutants relative to ACTIN (MADS-box gene nomenclature is based on [20]). Primers for the qRT-PCRs are listed in S1 Table. Bars represent averages of five replicates and error bars are s.e.m. Each replicate is a pool of six developing spikes at the early stamen primordia stage (W4.0 in the Waddington scale). ns = not significant, * = P < 0.05, ** = P < 0.01, and *** = P < 0.001. Data and statistical analyses used in E-G are available in the New Data D in S1 File. (TIF) [file pgen.1011096.s002.tif]
